# Pathway to Excellence Designation, Nurse Work Environment, and Hospital Quality and Safety: A Multi‐State Hospital Study

**DOI:** 10.1002/nur.70052

**Published:** 2026-01-07

**Authors:** Hyunmin Yu, J. Margo Brooks Carthon, Linda H. Aiken, Kevin K. McEwan, Matthew D. McHugh

**Affiliations:** ^1^ Center for Health Outcomes and Policy Research, School of Nursing University of Pennsylvania Philadelphia Pennsylvania USA; ^2^ Leonard Davis Institute of Health Economics University of Pennsylvania Philadelphia Pennsylvania USA; ^3^ Madisonhealth Rexburg Idaho USA

**Keywords:** Pathway to Excellence, patient safety, quality of care, work environment

## Abstract

The Pathway to Excellence (Pathway) program administered by the American Nurses Credentialing Center, recognizes organizations that foster positive nursing practice environments. However, evidence linking Pathway designation to nurse‐reported outcomes remains limited. This cross‐sectional study integrated data from the Penn 2024 Nurses4All Study and the American Hospital Association Annual Survey. The sample included 16,979 direct care nurses from 1672 hospitals in 10 US states (866 nurses in 63 Pathway hospitals and 16,113 nurses in 1609 non‐Pathway, non‐Magnet hospitals). The independent variable was Pathway status. Outcomes included nurse‐reported work environment, care quality, patient safety, and hospital recommendations. Multilevel linear and logistic regression models estimated associations. Pathway hospitals showed more favorable work environments (*γ*, the fixed‐effect coefficient from multilevel linear models = 0.08, 95% confidence interval [CI] = 0.01–0.16), driven by responsive administration (*γ* = 0.15, 95% CI = 0.05–0.24) and a clear philosophy of nursing (*γ* = 0.11, 95% CI = 0.01–0.20). Pathway hospitals also demonstrated a more favorable patient safety climate (*γ* = 0.51, 95% CI = 0.02–0.99), including more positive perceptions of a non‐punitive response to mistakes (*γ* = 0.12, 95% CI: 0.02–0.23), discussions about error prevention (*γ* = 0.12, 95% CI: 0.03–0.21), and leadership prioritization of safety (*γ* = 0.14, 95% CI: 0.02–0.26). Nurses in Pathway hospitals had higher odds of rating the overall work environment as excellent/good (adjusted odds ratio [aOR] = 1.32, 95% CI: 1.06–1.63), quality of nursing care as excellent/good (aOR = 1.35, 95% CI: 1.06–1.71), and of “definitely” recommending their hospital (aOR = 1.32, 95% CI: 1.03–1.69). These results underscore the central role of positive practice environments in delivering high‐quality, safer patient care.

## 1 Introduction

The American Nurses Credentialing Center (ANCC) administers both Magnet Recognition (Magnet) and Pathway to Excellence (Pathway), which are complementary yet distinct designations (Lal 2024). Magnet highlights institutions that consistently achieve superior patient care, foster nursing innovation, and uphold exemplary professional practice, with a strong emphasis on leadership, research, and measurable outcomes. Pathway, by contrast, centers on building and maintaining the fundamentals of a healthy practice environment in which nurses are engaged, empowered, and respected, prioritizing supportive workplace conditions and staff well‐being (Graystone and Pabico 2018).

The nurse work environment, defined as the organizational conditions that facilitate or impede professional nursing practice (Lake 2002), includes elements such as responsive leadership, adequate staffing, a clear nursing philosophy, and effective nurse–physician collaboration (Lake et al. 2024). Extensive research demonstrates that stronger work environments are linked to improved nurse job outcomes (Copanitsanou et al. 2017; Wei et al. 2018), higher quality of patient care (Lake et al. 2019; Lee and Scott 2018), and more favorable patient safety (Mihdawi et al. 2020). Given this evidence, promoting positive practice environments has become a central focus of nursing credentialing initiatives, including the Pathway program (Bates et al. 2020).

The Pathway program focuses on six foundational elements of an optimal nursing practice environment: (1) *shared decision‐making* that engages direct care nurses in collaboration and unit‐ and system‐level decisions, (2) *leadership* that enables shared governance through visible leaders who invest in development, retention, and succession, (3) *safety* that ensures a respectful, violence‐free workplace and protects both patients and staff, (4) *quality* that advances person‐ and family‐centered, evidence‐based care with continuous improvement, (5) *well‐being* that recognizes nurses and provides resources for physical and mental health, and (6) *professional development* that offers mentoring, support, and lifelong learning opportunities (Bates et al. 2020).

Despite the growing number of Pathway‐designated organizations, currently 277 facilities including 23 international organizations, as of December 2025 (American Nurses Credentialing Center 2025), evidence on the association between Pathway status and its core standards, including the work environment, quality, and safety, remains limited (Mulvey et al. 2023). Existing studies on Pathway have largely examined patient satisfaction (H. Yu, Golinelli, Aiken, et al. 2025; H. Yu, Golinelli, McHugh, et al. 2025). Understanding whether Pathway is associated with these outcomes is critical for assessing its practical value and identifying actionable levers for improvement.

### 1.1 Theoretical Framework

This study is grounded in Donabedian's quality of care framework, which conceptualizes healthcare quality as the relationship among *structure*, *process*, and *outcome* (Donabedian 2002). Within this framework, we treat Pathway designation as a structural attribute of hospitals, reflecting organizational investments in leadership accessibility, shared decision‐making, professional development, quality, safety, and well‐being. Structural features shape care processes, which include communication, teamwork, safety climate, and the overall practice environment. Accordingly, process measures such as the work environment, quality of nursing care, and patient safety climate represent the proximal manifestations of Pathway standards. This framework further posits that improved processes lead to more favorable outcomes, which guided our inclusion of nurses' likelihood to recommend the hospital as an engagement‐oriented outcome that reflects confidence in care quality and serves as an indirect indicator of care quality (Donabedian 2005). Therefore, this study aimed to explore the association between a hospital's Pathway status and (1) nurse‐reported work environments, (2) quality of nursing care, (3) patient safety climate, and (4) nurses' likelihood of recommending their hospital to family and friends.

## 2 Methods

### 2.1 Study Design and Data Sources

This cross‐sectional observational study integrated three data sources: the Penn 2024 Nurses4All Study, the 2023 American Hospital Association (AHA) Annual Survey, and the roster of Pathway‐designated organizations. The Nurses4All Study is a large, nurse‐reported survey on workplace conditions and environments. The 2024 wave captured nurse job outcomes and work environments across diverse healthcare settings in 10 states (CA, FL, IL, LA, NJ, NM, NY, OR, PA, and WA). In 2023 and 2024, nurses with active licenses in nine states were contacted by email using state licensure lists, while in Pennsylvania, where only mailing addresses were available, a 30% random sample received mailed invitations. Following a modified Dillman method, consistent with prior studies (Lasater et al. 2021, 2019; Stimpfel et al. 2016), we sent scheduled reminders to nonrespondents (Dillman et al. 2014).

Our previous work using a criterion‐standard double‐sampling method with intensive follow‐up of nonresponders has demonstrated minimal evidence of nonresponse bias with this survey strategy (Lasater et al. 2019). Moreover, our method of using nurses as informants and aggregating nurse responses to their employers eliminates response bias at the hospital level by including almost all hospitals of over 100 beds (Lasater et al. 2019). In addition, prior studies have validated that hospital‐level assessments of care quality based on modest numbers of nurse informants align closely with independent patient data (McHugh and Stimpfel 2012). Taken together, this evidence supports the validity of hospital‐level estimates even when the number of respondents per hospital is modest.

Because licensure lists do not indicate place of employment, the survey includes nurses working across multiple sectors, including hospitals, long‐term care, schools, and primary care, as well as nurses who are retired or unemployed. For this study, we restricted the sample to nurses employed in hospitals. Hospital attributes came from the 2023 AHA Annual Survey, which details hospital services, staffing, and operations (American Hospital Association 2023). Pathway status was identified from the ANCC's public listings (American Nurses Credentialing Center 2025). This study was approved by the University of Pennsylvania Institutional Review Board (protocol #850227) and conducted in accordance with institutional requirements and the ethical standards outlined in the Declaration of Helsinki. Data linkage was performed using unique hospital identifiers. The AHA number was used to link the Nurses4All with the AHA dataset. Each hospital in the merged dataset was then manually categorized by Pathway status using the ANCC's list of Pathway‐designated hospitals.

### 2.2 Study Sample

**Figure 1:**
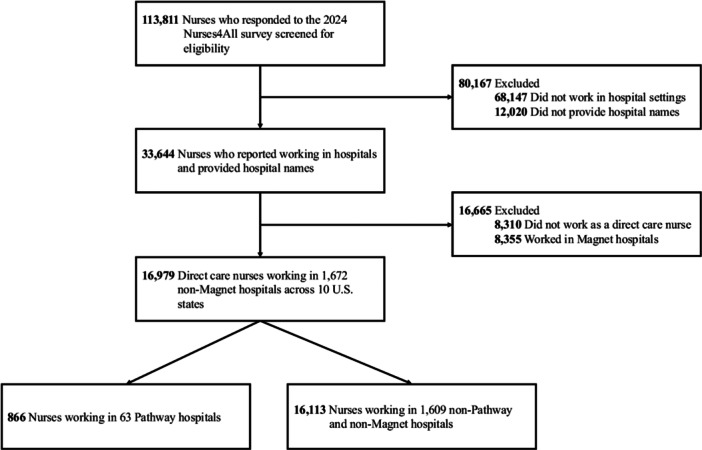
Subject inclusion diagram. This figure depicts the process of subject inclusion in the analysis.

To examine the association between hospitals' Pathway status and nurses' assessments of work environment, quality, and safety, we included respondents who (1) were employed in hospitals, (2) reported their hospital name to determine Pathway status, (3) did not work in Magnet hospitals, and (4) identified their role as direct care staff nurses. Although both programs are offered by the same accrediting body, Magnet and Pathway are separate recognitions that emphasize different dimensions of nursing excellence (Lal 2024; Pabico 2020). Because Magnet status is frequently linked to better work environments, quality, and safety (Rodríguez‐García et al. 2020), including Magnet hospitals could obscure estimates specific to the association between Pathway designation and outcomes. Given the different foci of Magnet and Pathway programs, we therefore excluded Magnet hospitals to ensure a focused comparison. Of the 113,811 nurses who completed the Penn 2024 Nurses4All survey, those not meeting these criteria were excluded. The final analytic sample comprised 16,979 direct care nurses from 1672 acute care hospitals across 10 US states, including 866 nurses in 63 Pathway hospitals and 16,113 nurses in 1609 non‐Pathway and non‐Magnet hospitals. These hospitals account for over 90% of all hospitals with 100 or more beds in the included states. The sampling flow is shown in Figure 1.

### 2.3 Study Variables

#### 2.3.1 Dependent Variable I: Work Environment

The nurse work environment was assessed with two measures. First, we used the validated short form of the Practice Environment Scale of the Nursing Work Index (PES‐5) (Lake et al. 2024). This instrument includes five items: (1) administration that listens and responds to nurses concerns, (2) physicians and nurses have good working relationships, (3) a supervisor who is a good manager and leader, (4) enough staff to get the work done, and (5) a clear philosophy of nursing that pervades the patient care environment. Each item was measured on a 4‐point Likert scale ranging from “1 = strongly agree” to “4 = strongly disagree.” All items were reverse‐coded so that higher scores reflected more favorable work environments. Cronbach's *α* was 0.80 in our sample. In primary analyses, we used the PES‐5 mean score; each of the five item scores was examined in sensitivity analyses.

We also included a single global item: “How would you rate the overall work environment of your primary job?” with response options: “1 = excellent,” “2 = good,” “3 = fair,” and “4 = poor.” For analysis, we dichotomized responses as 1 = more favorable (excellent/good) and 0 = less favorable (fair/poor).

#### 2.3.2 Dependent Variable II: Quality & Safety

The quality of nursing care was captured with a single validated item shown to correlate with objective patient outcomes, including mortality (McHugh and Stimpfel 2012; Smeds‐Alenius et al. 2016). Participants were asked, “In general, how would you describe the quality of nursing care delivered to patients in your work setting?” Responses used a 4‐point Likert scale with options of “1 = excellent,” “2 = good,” “3 = fair,” and “4 = poor” and were dichotomized for analysis, with excellent/good coded as 1 and fair/poor coded as 0 to distinguish favorable from less favorable ratings.

Nurses' likelihood of recommending their hospital was measured with the question, “Would you recommend where you work to your family and friends if they needed healthcare?” This measure, an indirect indicator of care quality that reflects provider preferences and behaviors (Donabedian 2005), has been validated internationally and aligns closely with patients' assessments of hospital quality (Aiken et al. 2012). Responses on a 4‐point scale (“1 = definitely yes,” “2 = probably yes,” “3 = probably not,” “4 = definitely not”) were recoded so that “definitely yes” = 1 and all other options = 0, isolating unequivocal endorsements from more uncertain or negative views.

Patient safety climate was assessed using the validated 7‐item Patient Safety Climate scale, a short form of the Hospital Survey on Patient Safety Culture (HSOPS), designed to reduce burden while retaining key organizational dimensions (Olds et al. 2017). The items included: (1) nurses feel like their mistakes are held against them, (2) nurses feel free to question the decisions or actions of those in authority, (3) we discuss ways to prevent errors from happening again, (4) important patient care information is often lost during shift changes or when another provider is covering my patients, (5) things “fall between the cracks” when transferring patients from one unit/care setting to another, (6) we are given feedback about changes put into place based on event reports, and (7) the actions of management show that patient safety is a top priority. Items were rated on a five‐point Likert scale (1 = strongly agree to 5 = strongly disagree). The four positively worded items (2, 3, 6, 7) were reverse‐coded so that higher values reflected a more favorable safety climate. Cronbach's *α* was 0.80 in our sample. Primary analyses used the summed total score, with item‐level analyses conducted as sensitivity checks.

#### 2.3.3 Independent Variable: Pathway Designation Status

The independent variable was hospitals' Pathway designation status, coded as a binary indicator: 0 for hospitals without Pathway designation and 1 for those with designation, based on 2024 status.

#### 2.3.4 Level 1 Covariates: Nurse Characteristics

We included a range of nurse characteristics in our models as level 1 covariates. The selected nurse characteristics were age, gender, educational attainment, and employment status, and they were chosen for their theoretical and empirical relevance to both Pathway status and these outcomes. Age can proxy experience and tenure, which may shape ratings of environment, quality, and safety (Van der Heijden et al. 2020). Workplace experiences may differ by gender (Göktepe and Sarıköse 2022). Educational attainment (highest nursing degree) influences role responsibilities and clinical judgment, potentially affecting quality and safety assessments (Aiken et al. 2011). Full‐time staff have greater exposure to the work environment and safety culture than part‐time staff. Age was modeled as a continuous variable. Gender included *male* or *female*. The highest nursing degree was categorized as *associate or lower*, *bachelor's*, or *graduate (master's and doctoral)*. Employment status included *full‐time* or *part‐time*.

#### 2.3.5 Level 2 Covariates: Hospital Characteristics

Various hospital characteristics were included as level 2 covariates. These were teaching status, size, and specialized service capacity. These variables were obtained from the AHA Annual Survey to account for structural factors that have been associated with work environment, quality, and safety (Aiken 2002). Teaching status was measured by the resident‐to‐bed ratio, considered more accurate than university affiliation (Ayanian et al. 1998) and categorized as *non‐teaching*, *minor teaching* (≤ 1 resident per 4 beds), or *major teaching* (> 1 per 4 beds). Hospital size was grouped as *small* (≤ 100 beds), *medium* (101–250 beds), or *large* (> 250 beds) (Aiken 2002). Specialized service capacity distinguished hospitals offering advanced procedures (e.g., open‐heart surgery or major organ transplants) as *high‐capacity*, with all others classified as *low‐capacity* (Hartz et al. 1989).

### 2.4 Statistical Analysis

Analyses were conducted in Stata (version 17) and R (version 4.5.1). We first used descriptive statistics to examine whether nurse and hospital characteristics and unadjusted outcomes differed by Pathway status. Because nurses (level 1) were nested within hospitals (level 2), we used multilevel linear models for continuous outcomes and multilevel logistic models for binary outcomes to account for the shared hospital context. Failing to model this clustering would violate the independence assumptions of ordinary regression and risk biased standard errors and misleading inferences (Austin and Merlo 2017; Sommet and Morselli 2017).

To investigate the effects of nurse‐level (level 1: age, gender, educational attainment, and employment status) and hospital‐level (level 2: Pathway status, teaching status, size, and specialized service capacity) factors on nurses' assessments of their work environment, care quality, and safety, we employed a two‐level random intercept model (Rabe‐Hesketh and Skrondal 2022). The random intercept allowed us to account for hospital‐level differences in these outcomes.

Our analytic process proceeded in several stages. We began by estimating an unconditional (null) model that included only the outcome and accounted for clustering by hospital. We then calculated the intraclass correlation coefficient (ICC) to determine how much of the variability in each outcome was attributable to differences between hospitals. After that, we specified a level 1 model containing nurse‐level predictors, followed by a full level 2 model that incorporated both nurse‐ and hospital‐level covariates (Peugh 2010). Prior to estimating each model, we evaluated multicollinearity using generalized variance inflation factors, with all values less than 1.6, indicating no multicollinearity concerns. For the multilevel linear models, regression diagnostics confirmed no violations of normality or heteroscedasticity. Additionally, likelihood‐ratio tests comparing the multilevel regression models with ordinary regression models were all statistically significant (*p* < 0.001), indicating that the inclusion of hospital‐level random effects significantly improved model fit.

Among the 16,979 direct care nurses who provided hospital identifiers, 9666 had complete data for all study variables, whereas 7313 had at least one missing value (43.1% missingness). Most missing responses were for age (*n* = 4543) and gender (*n *= 4359). Although the exact mechanism of missingness could not be determined, patterns in the data indicated that missingness was associated with observed characteristics, supporting the assumption of missing at random (MAR). Given this assumption and the substantial proportion of incomplete cases, using listwise deletion risked producing biased estimates (Allison 2010). To address this, we applied multiple imputation using multivariate imputation by chained equations (White et al. 2011) with the *mice* R package (Van Buuren and Groothuis‐Oudshoorn 2011).

## 3 Results

The overall nurse characteristics by hospitals' Pathway status are presented in Table 1. Among the 16,979 nurses, the average age was 46.2 years (SD = 12.7). Most were female (86.4%) and worked full‐time (82.8%). Over two‐thirds (67.3%) held a bachelor's degree or higher. Nurses in Pathway hospitals were younger than those in non‐Pathway hospitals (M = 45.3, SD = 12.7 vs. M = 46.3, SD = 12.7; *p* = 0.036).

**Table 1:** Nurse and hospital characteristics.

Nurse characteristics								
Characteristics	Total nurses (*N* = 16,979)		Nurses in Pathway hospitals (*n* = 866)		Nurses in non‐Pathway hospitals (*n* = 16,113)			
	M	SD	M	SD	M	SD	*t*	*p*
Age	46.2	12.7	45.3	12.7	46.3	12.7	−2.09	0.036
	***n***	**%**	***n***	**%**	***n***	**%**	***z***	***p***
Gender							1.05	0.295
Female	14,676	86.4	760	87.8	13,916	86.4		
Male	2303	13.6	106	12.2	2197	13.6		
Employment status							1.44	0.149
Full‐time	14,059	82.8	747	86.3	13,312	82.6		
Part‐time	2920	17.2	119	13.7	2801	17.4		
Highest nursing degree							0.12	0.908
Associate degree or lower	5555	32.7	298	34.4	5257	32.6		
Bachelor's degree or higher	11,424	67.3	568	65.6	10,856	67.4		

Hospital characteristics								
	Total hospitals (*N* = 1672)		Pathway hospitals (*n* = 63)		Non‐Pathway hospitals (*n* = 1609)			
	*n*	%	*n*	%	*n*	%	*z*	*p*
Hospital size							3.53	< 0.001
Up to 100 beds	724	43.3	13	20.6	711	44.2		
Over 100 beds	948	56.7	50	79.4	898	55.8		
Specialized services capacity							0.18	0.859
High	281	16.8	12	19.0	269	16.7		
Low	1391	83.2	51	81.0	1340	83.3		
Teaching status							−0.04	0.971
Non‐teaching	775	46.4	31	49.2	744	46.2		
Teaching (major and minor)	897	53.6	32	50.8	865	53.8		

Abbreviations: M = mean, SD = standard deviation.

Among the 1672 hospitals, more than half (56.7%) had over 100 beds, and the majority (83.2%) had low specialized service capacity (e.g., open‐heart surgery or major organ transplants). More than half (53.6%) were teaching hospitals. Pathway hospitals were more likely than non‐Pathway hospitals to have over 100 beds (79.4% vs. 55.8%; *p* < 0.001).

### 3.1 Unadjusted Outcome Differences by Pathway Status

Table 2 presents unadjusted outcome differences by Pathway status. Compared with nurses in non‐Pathway hospitals, those in Pathway hospitals more often rated the overall work environment as excellent/good (55.2% vs. 51.0%, *p* = 0.048). On specific PES‐5 items, they reported higher scores for “administration that listens and responds to nurse concerns” (2.41 vs. 2.29, *p* = 0.009). On patient safety climate items, Pathway hospitals scored higher for discussing ways to prevent errors (3.73 vs. 3.63, *p* = 0.009) and management actions showing that patient safety is a top priority (3.06 vs. 2.97, *p* = 0.045).

**Table 2:** Unadjusted outcome differences by Pathway status.

	Nurses in Pathway hospitals (*n* = 866)		Nurses in non‐Pathway hospitals (*n* = 16,113)				
Outcomes	M	SD	M	SD	*d*	*t*	*p*
Overall PES‐5 score^a^	2.68	0.73	2.63	0.71	0.069	1.51	0.131
Administration that listens and responds to nurse concerns^b^	2.41	0.98	2.29	0.98	0.122	2.62	0.009
Physicians and nurses have good working relationships	3.14	0.81	3.18	0.77	−0.050	−1.14	0.256
A supervisor who is a good manager and leader	2.84	1.04	2.80	1.03	0.039	1.13	0.260
Enough staff to get the work done	2.25	1.02	2.20	1.02	0.049	0.83	0.404
A clear philosophy of nursing that pervades the patient care environment	2.74	0.92	2.66	0.95	0.087	1.94	0.052
Total patient safety climate score^c^	21.18	5.28	20.84	5.07	0.065	1.81	0.070
Nurses feel like their mistakes are held against them^d^	2.57	1.16	2.52	1.12	0.044	1.76	0.078
Nurses feel free to question the decisions or actions of those in authority	2.98	1.20	2.95	1.17	0.026	1.05	0.292
We discuss ways to prevent errors from happening again	3.73	1.05	3.63	1.05	0.095	2.62	0.009
Important patient care information is often lost during shift changes or when another provider is covering my patients	2.84	1.12	2.84	1.09	0	−0.23	0.815
Things “fall between the cracks” when transferring patients from one unit/care setting to another	2.70	1.07	2.70	1.06	0	−0.51	0.609
We are given feedback about changes put into place based on event reports	3.31	1.17	3.23	1.18	0.069	1.81	0.070
The actions of management show that patient safety is a top priority	3.06	1.32	2.97	1.29	0.070	2.01	0.045
	***n***	**%**	***n***	**%**	**OR**	***z***	***p***
Overall work environment					1.18	1.98	0.048
Poor or fair	388	44.8	7895	49.0			
Excellent or good	478	55.2	8218	51.0			
Quality of nursing care					1.19	1.87	0.062
Poor or fair	218	25.2	4605	28.6			
Excellent or good	648	74.8	11,508	71.4			
Likelihood to recommend to family and friends					1.19	1.83	0.067
Definitely no, probably yes, probably no	648	74.8	12,564	78.0			
Definitely yes	218	25.2	3549	22.0			

Abbreviations: d = Cohen's d, M = mean, OR = (unadjusted) odds ratio, PES = practice environment scale, SD = standard deviation.

^a^ The mean PES‐5 score ranges from 1 to 4.

^b^ Each item from a validated short form of the Practice Environment Scale of the Nursing Work Index (PES‐5) ranges from 1 to 4, with higher scores indicating more positive aspects of work environment.

^c^ The total Patient Safety Climate score ranges from 7 to 35.

^d^ Each item from a validated short form of the Agency for Healthcare Research and Quality Hospital Survey on Patient Safety Culture (HSOPS) ranges from 1 to 5, with higher scores indicating more positive aspects of patient safety culture.

### 3.2 Multilevel Linear Model Results

The multilevel linear model includes a fixed‐effects component (gamma, *γ* coefficients) and a random‐effects component (tau, *τ* coefficients), which captures variability across hospital contexts (Paek et al. 2006). Supporting Information: Material 1 presents the full level 2 models for each continuous outcome (different aspects of the work environment and the patient safety climate), including the constant term (intercept), representing the overall average outcome across nurses assuming no hospital‐level differences; coefficients for all level 1 and level 2 predictors; random intercept variance, indicating variability across hospitals, and residual variance, indicating variability among nurses within the same hospital.

Table 3 presents multilevel linear regression estimates of nurses' assessments of different aspects of the work environment and the patient safety climate, along with ICC values indicating the proportion of variance attributable to differences between hospitals. Pathway status was associated with better overall work environment scores (*γ* = 0.08, 95% confidence interval [CI]: 0.01–0.16), driven by responsive administration (*γ* = 0.15, 95% CI: 0.05–0.24) and a clear philosophy of nursing (*γ* = 0.11, 95% CI: 0.01–0.20). Patient safety climate was more favorable overall (*γ* = 0.51, 95% CI: 0.02–0.99), with more positive perceptions of a non‐punitive response to mistakes (*γ* = 0.12, 95% CI: 0.02–0.23), discussions about error prevention (*γ* = 0.12, 95% CI: 0.03–0.21), and leadership prioritization of safety (*γ* = 0.14, 95% CI: 0.02–0.26).

**Table 3:** Multilevel linear regression estimates of nurses' assessments in Pathway hospitals.

	ICC	Estimate	95% CI	*p*
Overall PES‐5 score	0.10^a^	0.08	0.01–0.16	0.041*
Administration that listens and responds to nurses concerns	0.08	0.15	0.05–0.24	0.004**
Physicians and nurses have good working relationships	0.04	−0.02	−0.09–0.05	0.552
A supervisor who is a good manager and leader	0.04	0.07	−0.02–0.16	0.150
Enough staff to get the work done	0.13	0.09	−0.03–0.21	0.146
A clear philosophy of nursing that pervades the patient care environment	0.08	0.11	0.01–0.20	0.027*
Total patient safety climate score	0.06	0.51	0.02–0.99	0.041*
Nurses feel like their mistakes are held against them	0.05	0.12	0.02–0.23	0.017*
Nurses feel free to question the decisions or actions of those in authority	0.03	0.05	−0.05–0.14	0.320
We discuss ways to prevent errors from happening again	0.03	0.12	0.03–0.21	0.008**
Important patient care information is often lost during shift changes or when another provider is covering my patients	0.01	−0.01	−0.09–0.08	0.845
Things “fall between the cracks” when transferring patients from one unit/care setting to another	0.02	−0.01	−0.09–0.08	0.864
We are given feedback about changes put into place based on event reports	0.03	0.08	−0.02–0.18	0.111
The actions of management show that patient safety is a top priority	0.06	0.14	0.02–0.26	0.026*

*Note:* Multilevel models accounted for nurse‐level covariates (age, gender, employment status, and highest nursing degree) and hospital‐level covariates (hospital size, specialized service capacity, and teaching status).

Abbreviations: CI = confidence interval, ICC = intraclass correlation coefficient, PES = practice environment scale.

^a^ An ICC of 0.10 indicates that 10% of all variability in PES‐5 scores occurs at the hospital level.

* *p* < 0.05

** *p* < 0.01.

### 3.3 Multilevel Logistic Model Results

The multilevel logistic model also includes both fixed‐effects and random‐effects components. Supporting Information: Material 2 presents the full level 2 models for each binary outcome (the overall work environment, the quality of nursing care, and nurses' likelihood of recommending their hospital), including the constant term (intercept), which represents the average baseline odds of the outcome across nurses assuming no hospital‐level differences; adjusted odds ratios (aORs) for all level 1 and level 2 predictors; and the random intercept variance, which indicates variability in baseline odds across hospitals.

Table 4 shows multilevel logistic regression estimates of nurses' assessments of the overall work environment, the quality of nursing care, and their likelihood of recommending their hospital, along with ICC values indicating the proportion of variance attributable to differences between hospitals. Nurses in Pathway hospitals had higher odds of rating the overall work environment as excellent/good (aOR = 1.32, 95% CI: 1.06–1.63), the quality of nursing care as excellent/good (aOR = 1.35, 95% CI: 1.06–1.71), and of “definitely” recommending their hospital (aOR = 1.32, 95% CI: 1.03–1.69).

**Table 4:** Multilevel logistic regression estimates of nurses' assessments in Pathway hospitals.

	Overall work environment^a^				Quality of nursing care^a^				Likelihood to recommend^b^			
	ICC^c^	aOR	95% CI	*p*	ICC	aOR	95% CI	*p*	ICC	aOR	95% CI	*p*
Pathway status				0.013*				0.013*				0.026*
Yes	0.09	1.32	1.06; 1.63		0.10	1.35	1.06; 1.71		0.11	1.32	1.03; 1.69	

*Note:* Multilevel models accounted for nurse‐level covariates (age, gender, employment status, and highest nursing degree) and hospital‐level covariates (hospital size, specialized service capacity, and teaching status).

Abbreviations: aOR = adjusted odds ratio, CI = confidence interval, ICC = intraclass correlation coefficient.

* *p* < 0.05.

^a^ 0 = poor or fair, 1 = excellent or good.

^b^ 0 = definitely no, probably yes, probably no, 1 = definitely yes.

^c^ An ICC of 0.09 indicates that 9% of the variability in overall work environment ratings is explained by hospital‐level differences.

## 4 Discussion

This multi‐state study makes several contributions to the existing evidence on Pathway to Excellence (Pathway) designation. Prior research on Pathway has focused primarily on patient satisfaction (H. Yu, Golinelli, Aiken, et al. 2025; H. Yu, Golinelli, M. D. McHugh, et al. 2025), with limited examination of outcomes directly aligned with Pathway standards, such as nurse work environments, quality of nursing care, and patient safety climate. Our study provides the first evidence linking Pathway designation not only to more favorable nurse‐reported work environments but also to more positive assessments of care quality and safety climate, as well as a greater likelihood of recommending the hospital. These findings extend the growing evidence base on Pathway by demonstrating that the designation is associated with key proximal process outcomes, which are central to the Pathway program's aims, thereby offering empirical support for the program's foundational claims.

While effect sizes for some associations are modest, it is important to consider their practical significance at scale, the cumulative impact of small improvements across many units and shifts, and the likelihood that proximal, leadership‐sensitive indicators change earlier than distal outcomes such as turnover, patient complications, or cost. Even small, reliable shifts in responsive administration and error discussion processes can improve event reporting and accelerate organizational learning over time. Although the association between Pathway status and patient satisfaction during and after the COVID‐19 pandemic has been established (H. Yu, Golinelli, Aiken, et al. 2025; H. Yu, Golinelli, McHugh, et al. 2025), future research should test whether more favorable work environments, quality, and safety function as underlying mechanisms and evaluate downstream outcomes to determine whether these associations translate into sustained improvements in nurse well‐being and job outcomes, other patient clinical outcomes, and organizational performance.

Our findings align with prior evidence from home health settings showing that adherence to Pathway standards correlates with better nurse‐assessed quality and safety (Jarrín et al. 2017). The concept of the nurse work environment encompasses several elements, including responsive leadership, adequate staffing, a clear nursing philosophy, and effective nurse–physician collaboration (Lake et al. 2024). Pathway's emphasis on accessible leadership was reflected in significantly more favorable assessments of responsive administration and a clear nursing philosophy. However, elements such as staffing adequacy and physician–nurse relationships were not significantly different between groups. Although Pathway standards do not explicitly include domains such as staffing adequacy and physician−nurse relationships, these remain foundational to the nursing practice environment and should continue to be focal points in implementation and evaluation efforts linked to Pathway (Lake 2002). Both more favorable assessments of staffing adequacy (Turi et al. 2025) and stronger physician–nurse relationships (Labrague 2025) are associated with positive nurse and patient outcomes. Therefore, hospitals, regardless of Pathway status, should consider strengthening these organizational features to support nurse well‐being and patient outcomes.

Similarly, while Pathway hospitals demonstrated an overall more favorable nurse‐reported patient safety climate, they did not outperform non‐Pathway hospitals across all aspects. As with the work environment findings, item‐level analyses showed that the strongest and most consistent advantages in Pathway hospitals clustered around administration and managerial actions, including discussing error prevention, prioritizing safety at the leadership level, and ensuring that mistakes are not held against nurses. However, dimensions related to questioning authority, loss of information during transfers or shift changes, and feedback from incident reports were not significantly different between groups, despite their importance for patient and nurse outcomes (Chen et al. 2024; de Lisser et al. 2024; Mardis et al. 2017). These elements can be strengthened by fostering psychological safety at work. Psychological safety in teams refers to a shared belief that members can take interpersonal risks, including speaking up, without harm (Edmondson 1999). It involves creating conditions in which individuals feel comfortable raising questions or concerns without judgment or repercussions. Psychological safety has also been linked to lower levels of nurse burnout (de Lisser et al. 2024; Kerrissey et al. 2022). It can be strengthened through leadership behaviors (e.g., modeling fallibility, inviting dissent, closing the loop on concerns) and through professional‐governance forums such as unit councils, chief nursing officer town halls (Riesch et al. 2023), and employee resource groups (Seegmiller Renner et al. 2022) that create regular, low‐risk channels to speak up. These approaches enhance transparency, elevate frontline voices, and establish consistent avenues for speaking up, fostering a stronger safety culture.

Sharing follow‐up changes from event reports is also critical. Existing healthcare reporting systems, such as adverse event reporting (Falcone et al. 2022) or incident reporting (Campbell 2017), have often prioritized collecting reports rather than learning from reported events and addressing root issues, which contrasts with high‐risk industries such as aviation (Macrae 2016). Although adverse event reporting has been associated with increased job satisfaction and reduced burnout (Chen et al. 2024), studies highlight widespread underreporting by both those who directly experience the issue (Gianakos et al. 2022; Phillips 2016) and those who witness it (Milliken et al. 2003; H. H. Yu 2024). Barriers to reporting include an organizational culture of blame (Weiner et al. 2008), fear of repercussions because most reports are reviewed directly by leadership (Hewitt et al. 2016; Weiner et al. 2008; Yusuf and Irwan 2021), and expectations that no meaningful changes will result or that feedback on incident follow‐up will be inadequate (Arnetz et al. 2015; Evans et al. 2006). Because employee silence can significantly undermine organizational learning and contribute to unresolved systemic issues (Morrison 2023), hospitals should work to reduce these barriers by consistently sharing feedback about actions taken in response to event reports.

### 4.1 Strengths and Limitations

This study's strengths are the inclusion of almost all hospitals in 10 US states and the use of multilevel modeling to rigorously account for both nurse‐ and hospital‐level covariates. Several limitations warrant consideration. First, the cross‐sectional design precludes causal inference. Second, a binary Pathway indicator lacks nuance: it excludes “on‐the‐journey” hospitals, obscures dose‐response by time since designation or fidelity, and cannot capture cumulative effects of multiple Pathway re‐designations. Third, although our prior work using a rigorous double‐sampling method with extensive outreach to nonresponders has found minimal evidence of nonresponse bias with this survey approach (Lasater et al. 2019), the number of nurse respondents per hospital in this study was modest, which may limit the precision of hospital‐level estimates.

## 5 Conclusions

Pathway to Excellence designation was associated with more favorable work environments, higher quality of nursing care, a stronger patient safety climate, and a greater likelihood of nurses recommending the hospital, underscoring the central role of positive practice environments in delivering high‐quality and safe care. Supportive conditions that elevate clinician engagement, enable responsive leadership, and sustain professional development create the processes through which structure translates into better outcomes for patients and clinicians. Investing in practice environments supportive of excellent care is therefore not only an organizational imperative for nurse retention and well‐being, but also a strategic pathway to safer patient care and sustained organizational performance.

## Author Contributions

All authors made contributions to the conception and design of this article. All authors contributed to the acquisition, analysis, and interpretation of data. H.Y. drafted the manuscript. H.Y., J.M.B.C., L.H.A., K.K.M., and M.D.M. revised the manuscript. All authors approved the final manuscript.

## Conflicts of Interest

The authors declare no conflicts of interest.

## Supporting information

supmat.

## Data Availability

Research data are not shared.

## References

[nur70052-bib-0001] Aiken, L. H. 2002. “Hospital Nurse Staffing and Patient Mortality, Nurse Burnout, and Job Dissatisfaction.” Journal of the American Medical Association 288, no. 16: 1987–1993. 10.1001/jama.288.16.1987. 12387650

[nur70052-bib-0002] Aiken, L. H. , J. P. Cimiotti , D. M. Sloane , H. L. Smith , L. Flynn , and D. F. Neff . 2011. “Effects of Nurse Staffing and Nurse Education on Patient Deaths in Hospitals With Different Nurse Work Environments.” Medical Care 49, no. 12: 1047–1053. 10.1097/MLR.0b013e3182330b6e. 21945978 PMC3217062

[nur70052-bib-0003] Aiken, L. H. , W. Sermeus , K. Van den Heede , et al. 2012. “Patient Safety, Satisfaction, and Quality of Hospital Care: Cross Sectional Surveys of Nurses and Patients in 12 Countries in Europe and the United States.” BMJ 344: e1717. 10.1136/bmj.e1717. 22434089 PMC3308724

[nur70052-bib-0004] Allison, P. D. 2010. “Missing Data.” In The SAGE Handbook of Quantitative Methods in Psychology, edited by R. E. Millsap and A. Maydeu‐Olivares , 632–657. SAGE Publications.

[nur70052-bib-0005] American Hospital Association . 2024. “2023 AHA Annual Survey.” https://www.ahadata.com/system/files/media/file/2024/09/AHA‐Annual‐Survey‐2023.pdf.

[nur70052-bib-0006] American Nurses Credentialing Center . 2025. “Find a Pathway Organization.” https://www.nursingworld.org/organizational‐programs/pathway/find‐a‐pathway‐organization/.

[nur70052-bib-0007] Arnetz, J. E. , L. Hamblin , J. Ager , et al. 2015. “Underreporting of Workplace Violence: Comparison of Self‐Report and Actual Documentation of Hospital Incidents.” Workplace Health & Safety 63, no. 5: 200–210. 10.1177/2165079915574684. 26002854 PMC5006066

[nur70052-bib-0008] Austin, P. C. , and J. Merlo . 2017. “Intermediate and Advanced Topics in Multilevel Logistic Regression Analysis.” Statistics in Medicine 36, no. 20: 3257–3277. 10.1002/sim.7336. 28543517 PMC5575471

[nur70052-bib-0009] Ayanian, J. Z. , J. S. Weissman , S. Chasan‐Taber , and A. M. Epstein . 1998. “Quality of Care for Two Common Illnesses in Teaching and Nonteaching Hospitals.” Health Affairs 17, no. 6: 194–205. 10.1377/hlthaff.17.6.194. 9916369

[nur70052-bib-0010] Bates, M. , J. Hargreaves , M. McCright , C. Pabico , and L. Hume . 2020. “Introducing the 2020 Pathway to Excellence Manual.” Nursing Management 51, no. 4: 7–10. 10.1097/01.NUMA.0000657296.39677.a6. 32221121

[nur70052-bib-0011] Campbell, C. L. 2017. “Incident Reporting by Health‐Care Workers in Noninstitutional Care Settings.” Trauma, Violence & Abuse 18, no. 4: 445–456. 10.1177/1524838015627148. 26762136

[nur70052-bib-0012] Chen, Y. , Y. He , P. Wang , et al. 2024. “The Association Between the Adverse Event Reporting System and Burnout and Job Satisfaction of Nurses: Workplace Violence as a Mediator.” International Nursing Review 71, no. 4: 1053–1061. 10.1111/inr.12962. 38650586

[nur70052-bib-0013] Copanitsanou, P. , N. Fotos , and H. Brokalaki . 2017. “Effects of Work Environment on Patient and Nurse Outcomes.” British Journal of Nursing 26, no. 3: 172–176. 10.12968/bjon.2017.26.3.172. 28185485

[nur70052-bib-0014] de Lisser, R. , M. S. Dietrich , J. Spetz , R. Ramanujam , J. Lauderdale , and D. P. Stolldorf . 2024. “Psychological Safety Is Associated With Better Work Environment and Lower Levels of Clinician Burnout.” Health Affairs Scholar 2, no. 7: qxae091. 10.1093/haschl/qxae091. 39081721 PMC11288325

[nur70052-bib-0015] Dillman, D. A. , J. D. Smyth , and L. M. Christian . 2014. Internet, Phone, Mail, and Mixed‐Mode Surveys: The Tailored Design Method. 4th ed. John Wiley & Sons Inc.

[nur70052-bib-0016] Donabedian, A. 2002. An Introduction to Quality Assurance in Health Care. Oxford University Press.

[nur70052-bib-0017] Donabedian, A. 2005. “Evaluating the Quality of Medical Care.” Milbank Quarterly 83, no. 4: 691–729. 10.1111/j.1468-0009.2005.00397.x. 16279964 PMC2690293

[nur70052-bib-0018] Edmondson, A. 1999. “Psychological Safety and Learning Behavior in Work Teams.” Administrative Science Quarterly 44, no. 2: 350–383. 10.2307/2666999.

[nur70052-bib-0019] Evans, S. M. , J. G. Berry , B. J. Smith , et al. 2006. “Attitudes and Barriers to Incident Reporting: A Collaborative Hospital Study.” Quality & Safety in Health Care 15, no. 1: 39–43. 10.1136/qshc.2004.012559. 16456208 PMC2563993

[nur70052-bib-0020] Falcone, M. L. , S. K. Van Stee , U. Tokac , and A. F. Fish . 2022. “Adverse Event Reporting Priorities: An Integrative Review.” Journal of Patient Safety 18, no. 4: e727–e740. 10.1097/PTS.0000000000000945. 35617598

[nur70052-bib-0021] Gianakos, A. L. , J. A. Freischlag , A. M. Mercurio , et al. 2022. “Bullying, Discrimination, Harassment, Sexual Harassment, and the Fear of Retaliation During Surgical Residency Training: A Systematic Review.” World Journal of Surgery 46, no. 7: 1587–1599. 10.1007/s00268-021-06432-6. 35006329

[nur70052-bib-0022] Göktepe, N. , and S. Sarıköse . 2022. “Same Place but Different Experience: A Qualitative Study on Gender and the Nursing Work Environment.” Journal of Nursing Management 30, no. 7: 3227–3235. 10.1111/jonm.13748. 35895493

[nur70052-bib-0023] Graystone, R. , and C. Pabico . 2018. “Magnet or Pathway Recognition: Comparing and Contrasting.” JONA: Journal of Nursing Administration 48, no. 5: 233–234. 10.1097/NNA.0000000000000606. 29672370

[nur70052-bib-0024] Hartz, A. J. , H. Krakauer , E. M. Kuhn , et al. 1989. “Hospital Characteristics and Mortality Rates.” New England Journal of Medicine 321, no. 25: 1720–1725. 10.1056/NEJM198912213212506. 2594031

[nur70052-bib-0025] Hewitt, T. , S. Chreim , and A. Forster . 2016. “Incident Reporting Systems: A Comparative Study of Two Hospital Divisions.” Archives of Public Health 74: 34. 10.1186/s13690-016-0146-8. 27529024 PMC4983791

[nur70052-bib-0026] Jarrín, O. F. , Y. Kang , and L. H. Aiken . 2017. “Pathway to Better Patient Care and Nurse Workforce Outcomes in Home Care.” Nursing Outlook 65, no. 6: 671–678. 10.1016/j.outlook.2017.05.009. 28662969 PMC5712278

[nur70052-bib-0027] Kerrissey, M. J. , T. C. Hayirli , A. Bhanja , N. Stark , J. Hardy , and C. R. Peabody . 2022. “How Psychological Safety and Feeling Heard Relate to Burnout and Adaptation Amid Uncertainty.” Health Care Management Review 47, no. 4: 308–316. 10.1097/HMR.0000000000000338. 35135989 PMC9422764

[nur70052-bib-0028] Labrague, L. J. 2025. “A Systematic Review on Nurse‐Physician Collaboration and Its Relationship With Nursing Workforce Outcomes: Implications for Nursing Administration.” JONA: Journal of Nursing Administration 55, no. 3: 157–164. 10.1097/NNA.0000000000001549. 39970026

[nur70052-bib-0029] Lake, E. T. 2002. “Development of the Practice Environment Scale of the Nursing Work Index.” Research in Nursing & Health 25, no. 3: 176–188. 10.1002/nur.10032. 12015780

[nur70052-bib-0030] Lake, E. T. , J. Gil , L. Moronski , M. D. Mchugh , L. H. Aiken , and K. B. Lasater . 2024. “Validation of a Short Form of the Practice Environment Scale of the Nursing Work Index: The PES‐5.” Research in Nursing & Health 47, no. 4: 450–459. 10.1002/nur.22388. 38669131 PMC11236491

[nur70052-bib-0031] Lake, E. T. , J. Sanders , R. Duan , K. A. Riman , K. M. Schoenauer , and Y. Chen . 2019. “A Meta‐Analysis of the Associations Between the Nurse Work Environment in Hospitals and 4 Sets of Outcomes.” Medical Care 57, no. 5: 353–361. 10.1097/MLR.0000000000001109. 30908381 PMC6615025

[nur70052-bib-0032] Lal, M. M. 2024. “Magnet Recognition and Pathway to Excellence, Complementary Programs.” JONA: Journal of Nursing Administration 54, no. 2: 67–68. 10.1097/NNA.0000000000001381. 38261636

[nur70052-bib-0033] Lasater, K. B. , L. H. Aiken , D. M. Sloane , et al. 2021. “Is Hospital Nurse Staffing Legislation in the Public's Interest?: An Observational Study in New York State.” Medical Care 59, no. 5: 444–450. 10.1097/MLR.0000000000001519. 33655903 PMC8026733

[nur70052-bib-0034] Lasater, K. B. , O. F. Jarrín , L. H. Aiken , M. D. McHugh , D. M. Sloane , and H. L. Smith . 2019. “A Methodology for Studying Organizational Performance: A Multistate Survey of Front‐Line Providers.” Medical Care 57, no. 9: 742–749. 10.1097/MLR.0000000000001167. 31274782 PMC6690788

[nur70052-bib-0035] Lee, S. E. , and L. D. Scott . 2018. “Hospital Nurses' Work Environment Characteristics and Patient Safety Outcomes: A Literature Review.” Western Journal of Nursing Research 40, no. 1: 121–145. 10.1177/0193945916666071. 27586440

[nur70052-bib-0036] Macrae, C. 2016. “The Problem With Incident Reporting.” BMJ Quality & Safety 25, no. 2: 71–75. 10.1136/bmjqs-2015-004732. 26347519

[nur70052-bib-0037] Mardis, M. , J. Davis , B. Benningfield , et al. 2017. “Shift‐to‐Shift Handoff Effects on Patient Safety and Outcomes: A Systematic Review.” American Journal of Medical Quality 32, no. 1: 34–42. 10.1177/1062860615612923. 26518882

[nur70052-bib-0038] McHugh, M. D. , and A. W. Stimpfel . 2012. “Nurse Reported Quality of Care: A Measure of Hospital Quality.” Research in Nursing & Health 35, no. 6: 566–575. 10.1002/nur.21503. 22911102 PMC3596809

[nur70052-bib-0039] Mihdawi, M. , R. Al‐Amer , R. Darwish , S. Randall , and T. Afaneh . 2020. “The Influence of Nursing Work Environment on Patient Safety.” Workplace Health & Safety 68, no. 8: 384–390. 10.1177/2165079920901533. 32193998

[nur70052-bib-0040] Milliken, F. J. , E. W. Morrison , and P. F. Hewlin . 2003. “An Exploratory Study of Employee Silence: Issues That Employees Don't Communicate Upward and Why.” Journal of Management Studies 40, no. 6: 1453–1476. 10.1111/1467-6486.00387.

[nur70052-bib-0041] Morrison, E. W. 2023. “Employee Voice and Silence: Taking Stock a Decade Later.” Annual Review of Organizational Psychology and Organizational Behavior 10, no. 1: 79–107. 10.1146/annurev-orgpsych-120920-054654.

[nur70052-bib-0042] Mulvey, T. , M. Cámpoli , and V. Lundmark . 2023. “Establishing a Pathway to Excellence Research Agenda: A Delphi Study to Identify Research Priorities for Evaluating Positive Practice Environments.” JONA: Journal of Nursing Administration 53, no. 4: 189–196. 10.1097/NNA.0000000000001269. PMC10026953 36916787

[nur70052-bib-0064] Olds, D. M. , L. H. Aiken , J. P. Cimiotti , and E. T. Lake . 2017. “Association of Nurse Work Environment and Safety Climate on Patient Mortality: A Cross‐Sectional Study.” International Journal of Nursing Studies 74: 155–161. 10.1016/j.ijnurstu.2017.06.004. 28709013 PMC5695880

[nur70052-bib-0043] Pabico, C. 2020. “Dispelling Common Myths about ANCC's Pathway to Excellence.” American Nurse Journal 15, no. 5: 34–37.

[nur70052-bib-0044] Paek, H.‐J. , B. Lee , C. T. Salmon , and K. Witte . 2008. “The Contextual Effects of Gender Norms, Communication, and Social Capital on Family Planning Behaviors in Uganda: A Multilevel Approach.” Health Education & Behavior: The Official Publication of the Society for Public Health Education 35, no. 4: 461–477. 10.1177/1090198106296769. 17513691

[nur70052-bib-0045] Peugh, J. L. 2010. “A Practical Guide to Multilevel Modeling.” Journal of School Psychology 48, no. 1: 85–112. 10.1016/j.jsp.2009.09.002. 20006989

[nur70052-bib-0046] Phillips, J. P. 2016. “Workplace Violence Against Health Care Workers in the United States.” New England Journal of Medicine 374, no. 17: 1661–1669. 10.1056/NEJMra1501998. 27119238

[nur70052-bib-0047] Rabe‐Hesketh, S. , and A. Skrondal . 2022. Multilevel and Longitudinal Modeling Using Stata. 4th ed. STATA Press.

[nur70052-bib-0048] Riesch, S. K. , J. Chiappa , N. Floyd , and M. Ponce . 2023. “The Chief Nursing Officer Shared Leadership Model.” Nurse Leader 21, no. 1: 31–37. 10.1016/j.mnl.2022.09.006.

[nur70052-bib-0049] Rodríguez‐García, M. C. , V. V. Márquez‐Hernández , T. Belmonte‐García , L. Gutiérrez‐Puertas , and G. Granados‐Gámez . 2020. “Original Research: How Magnet Hospital Status Affects Nurses, Patients, and Organizations: A Systematic Review.” AJN, American Journal of Nursing 120, no. 7: 28–38. 10.1097/01.NAJ.0000681648.48249.16. 32541337

[nur70052-bib-0050] Seegmiller Renner, A. M. , H. L. Borgwardt , M. Coyle , S. Moeschler , and A. Bhagra . 2022. “Using an Employee Resource Group to Develop Grit in Female Healthcare Leaders: A Case Study.” Leadership in Health Services 35, no. 2: 267–284. 10.1108/LHS-04-2021-0028. 34978393

[nur70052-bib-0051] Smeds‐Alenius, L. , C. Tishelman , R. Lindqvist , S. Runesdotter , and M. D. McHugh . 2016. “RN Assessments of Excellent Quality of Care and Patient Safety Are Associated With Significantly Lower Odds of 30‐Day Inpatient Mortality: A National Cross‐Sectional Study of Acute‐Care Hospitals.” International Journal of Nursing Studies 61: 117–124. 10.1016/j.ijnurstu.2016.06.005. 27348357 PMC5072172

[nur70052-bib-0052] Sommet, N. , and D. Morselli . 2017. “Keep Calm and Learn Multilevel Logistic Modeling: A Simplified Three‐Step Procedure Using Stata, R, Mplus, and SPSS.” International Review of Social Psychology 30: 203–218. 10.5334/irsp.90.

[nur70052-bib-0053] Stimpfel, A. W. , D. M. Sloane , M. D. McHugh , and L. H. Aiken . 2016. “Hospitals Known for Nursing Excellence Associated With Better Hospital Experience for Patients.” Health Services Research 51, no. 3: 1120–1134. 10.1111/1475-6773.12357. 26369862 PMC4874824

[nur70052-bib-0054] Turi, E. , K. B. Lasater , and K. J. Muir . 2025. “Nurse Staffing and Patient Outcomes: A Call to Action for Chronic Wound Care Policy Reform.” Advances in Wound Care, ahead of print, December 19. 10.1177/21621918251400770. PMC13192300 41309220

[nur70052-bib-0055] Van Buuren, S. , and K. Groothuis‐Oudshoorn . 2011. “Mice: Multivariate Imputation by Chained Equations in R.” Journal of Statistical Software 45: 1–67. 10.18637/jss.v045.i03.

[nur70052-bib-0056] Van der Heijden, B. I. J. M. , I. Houkes , A. Van den Broeck , and K. Czabanowska . 2020. “‘I Just Can't Take It Anymore’: How Specific Work Characteristics Impact Younger Versus Older Nurses' Health, Satisfaction, and Commitment.” Frontiers in Psychology 11: 762. 10.3389/fpsyg.2020.00762. 32536884 PMC7267024

[nur70052-bib-0057] Wei, H. , K. A. Sewell , G. Woody , and M. A. Rose . 2018. “The State of the Science of Nurse Work Environments in the United States: A Systematic Review.” International Journal of Nursing Sciences 5, no. 3: 287–300. 10.1016/j.ijnss.2018.04.010. 31406839 PMC6626229

[nur70052-bib-0058] Weiner, B. J. , C. Hobgood , and M. A. Lewis . 2008. “The Meaning of Justice in Safety Incident Reporting.” Social Science & Medicine (1982) 66, no. 2: 403–413. 10.1016/j.socscimed.2007.08.013. 17949876

[nur70052-bib-0059] White, I. R. , P. Royston , and A. M. Wood . 2011. “Multiple Imputation Using Chained Equations: Issues and Guidance for Practice.” Statistics in Medicine 30, no. 4: 377–399. 10.1002/sim.4067. 21225900

[nur70052-bib-0060] Yu, H. , D. Golinelli , L. H. Aiken , M. D. McHugh , and J. M. Brooks Carthon . 2025. “The Protective Effect of Pathway to Excellence Designation on Patient Satisfaction During the COVID‐19 Pandemic.” International Journal for Quality in Health Care 37, no. 4: mzaf096. 10.1093/intqhc/mzaf096. 40973077

[nur70052-bib-0061] Yu, H. , D. Golinelli , M. D. McHugh , J. M. Brooks Carthon , and L. H. Aiken . 2025. “The Influence of Pathway to Excellence Designation on Patient Satisfaction.” JONA: Journal of Nursing Administration 55, no. 8: 457–464. 10.1097/NNA.0000000000001610. 40844537

[nur70052-bib-0062] Yu, H. H. 2024. “Reporting Workplace Discrimination: An Exploratory Analysis of Bystander Behavior.” Review of Public Personnel Administration 44, no. 3: 453–471. 10.1177/0734371X221149164.

[nur70052-bib-0063] Yusuf, Y. , and A. M. Irwan . 2021. “The Influence of Nurse Leadership Style on the Culture of Patient Safety Incident Reporting: A Systematic Review.” British Journal of Healthcare Management 27, no. 6: 1–7. 10.12968/bjhc.2020.0083.

