# Supplementary material for: Pathway to Excellence Designation, Nurse Work Environment, and Hospital Quality and Safety: A Multi‐State Hospital Study
Source: Res Nurs Health. 2026 Jan 7;49(2):159–69. doi: 10.1002/nur.70052 (PMC12954649; doi:10.1002/nur.70052)
Supplement: Supplementary file 1 — supmat. [file NUR-49-159-s001.docx]

**Supplemental Material 1**. Multilevel Linear Regression Results for Nurse Work Environment and Patient Safety Climate

|  | **Overall PES-5 Score** ^a^ | | | **Total Patient Safety Climate Score** ^b^ | | |
| --- | --- | --- | --- | --- | --- | --- |
|  | **Estimate** | **95% CI** | ***p*** | **Estimate** | **95% CI** | ***p*** |
| **Intercept** | 2.45 | 2.39; 2.51 | <0.001*** | 19.78 | 19.36; 20.20 | <0.001*** |
| **Fixed effects: Nurse-level variables** |  |  |  |  |  |  |
| **Age** | 0.01 | 0.01; 0.01 | <0.001*** | 0.02 | 0.01; 0.02 | <0.001*** |
| **Highest nursing degree** |  |  |  |  |  |  |
| Associate degree or lower | -0.03 | -0.05; -0.01 | 0.032* | -0.25 | -0.42; -0.08 | 0.004** |
| Bachelor’s degree (reference) |  |  |  |  |  |  |
| Graduate degree | -0.07 | -0.11; -0.04 | <0.001*** | -0.61 | -0.88; -0.34 | <0.001*** |
| **Gender** |  |  |  |  |  |  |
| Female (reference) |  |  |  |  |  |  |
| Male | 0.02 | -0.01; 0.05 | 0.121 | -0.22 | -0.44; 0.01 | 0.054 |
| **Employment status** |  |  |  |  |  |  |
| Full-time (reference) |  |  |  |  |  |  |
| Part-time | 0.01 | -0.02; 0.04 | 0.362 | 0.21 | 0.01; 0.41 | 0.049* |
| **Fixed effects: Hospital-level variables** |  |  |  |  |  |  |
| **Pathway status** |  |  |  |  |  |  |
| Yes | 0.08 | 0.01; 0.16 | 0.041* | 0.51 | 0.02; 0.99 | 0.041* |
| No (reference) |  |  |  |  |  |  |
| **Hospital size** |  |  |  |  |  |  |
| Small (up to 100 beds) | 0.21 | 0.16; 0.26 | <0.001*** | 1.08 | 0.75; 1.39 | <0.001*** |
| Medium (101-250 beds) | 0.07 | 0.03; 0.12 | 0.001** | 0.43 | 0.17; 0.70 | 0.001** |
| Large (over 250 beds) (reference) |  |  |  |  |  |  |
| **Teaching status** |  |  |  |  |  |  |
| None (reference) |  |  |  |  |  |  |
| Minor | -0.04 | -0.08; -0.01 | 0.031* | -0.27 | -0.51; -0.03 | 0.031* |
| Major | 0.02 | -0.04; 0.08 | 0.471 | 0.51 | 0.14; 0.87 | 0.007** |
| **Specialized service capacity** |  |  |  |  |  |  |
| High capacity | 0.06 | 0.01; 0.10 | 0.008** | 0.25 | -0.01; 0.51 | 0.061 |
| Low capacity (reference) |  |  |  |  |  |  |
| **Random effects** |  |  |  |  |  |  |
| Random intercept variance | 0.05 | 0.04; 0.05 | <0.001*** | 1.41 | 1.14; 1.73 | <0.001*** |
| Residual variance | 0.45 | 0.44; 0.46 | <0.001*** | 24.20 | 23.67; 24.74 | <0.001*** |

Abbreviation: CI = confidence interval, PES = practice environment scale

* *p*<0.05; ** *p*<0.01; *** *p*<0.001

a: The mean PES-5 score ranges from 1 to 4.

b: The total Patient Safety Climate score ranges from 6 to 30.

**Supplemental Material 2**. Multilevel Logistic Regression Results for Overall Work Environment, Quality of Nursing Care, and Likelihood of Hospital Recommendation

|  | **Overall Work Environment** ^a^ | | | **Quality of Nursing Care** ^a^ | | | **Likelihood to Recommend** ^b^ | | |
| --- | --- | --- | --- | --- | --- | --- | --- | --- | --- |
|  | **aOR** | **95% CI** | ***p*** | **aOR** | **95% CI** | ***p*** | **aOR** | **95% CI** | ***p*** |
| **Intercept** | 0.52 | 0.44; 0.63 | <0.001*** | 0.90 | 0.74; 1.10 | 0.305 | 0.09 | 0.07; 0.11 | <0.001*** |
| **Fixed effects: Nurse-level variables** |  |  |  |  |  |  |  |  |  |
| **Age** | 1.01 | 1.00; 1.01 | <0.001*** | 1.02 | 1.01; 1.02 | <0.001*** | 1.02 | 1.01; 1.02 | <0.001*** |
| **Highest nursing degree** |  |  |  |  |  |  |  |  |  |
| Associate degree or lower | 0.91 | 0.85; 0.98 | 0.009** | 0.88 | 0.82; 0.96 | 0.002** | 0.89 | 0.82; 0.97 | 0.011* |
| Bachelor’s degree (reference) |  |  |  |  |  |  |  |  |  |
| Graduate degree | 0.98 | 0.88; 1.10 | 0.779 | 0.87 | 0.77; 0.99 | 0.029* | 0.99 | 0.87; 1.14 | 0.961 |
| **Gender** |  |  |  |  |  |  |  |  |  |
| Female (reference) |  |  |  |  |  |  |  |  |  |
| Male | 1.07 | 0.97; 1.17 | 0.183 | 0.84 | 0.76; 0.93 | 0.001** | 0.91 | 0.81; 1.02 | 0.091 |
| **Employment status** |  |  |  |  |  |  |  |  |  |
| Full-time (reference) |  |  |  |  |  |  |  |  |  |
| Part-time | 1.09 | 1.00; 1.19 | 0.049* | 1.19 | 1.08; 1.32 | 0.001** | 1.12 | 1.01; 1.24 | 0.029* |
| **Fixed effects: Hospital-level variables** |  |  |  |  |  |  |  |  |  |
| **Pathway status** |  |  |  |  |  |  |  |  |  |
| Yes | 1.32 | 1.06; 1.63 | 0.013* | 1.35 | 1.06; 1.71 | 0.013* | 1.32 | 1.03; 1.69 | 0.026* |
| No (reference) |  |  |  |  |  |  |  |  |  |
| **Hospital size** |  |  |  |  |  |  |  |  |  |
| Small (up to 100 beds) | 1.61 | 1.40; 1.86 | <0.001*** | 1.82 | 1.56; 2.13 | <0.001*** | 1.50 | 1.27; 1.77 | <0.001*** |
| Medium (101-250 beds) | 1.23 | 1.09; 1.38 | 0.001** | 1.26 | 1.11; 1.43 | <0.001*** | 1.05 | 0.91; 1.21 | 0.491 |
| Large (over 250 beds) (reference) |  |  |  |  |  |  |  |  |  |
| **Teaching status** |  |  |  |  |  |  |  |  |  |
| None (reference) |  |  |  |  |  |  |  |  |  |
| Minor | 0.95 | 0.85; 1.05 | 0.313 | 0.95 | 0.85; 1.07 | 0.408 | 0.83 | 0.73; 0.94 | 0.004** |
| Major | 1.11 | 0.94; 1.30 | 0.225 | 1.20 | 1.01; 1.43 | 0.040* | 1.01 | 0.84; 1.22 | 0.908 |
| **Specialized service capacity** |  |  |  |  |  |  |  |  |  |
| High capacity | 1.21 | 1.08; 1.36 | 0.001** | 1.25 | 1.10; 1.41 | <0.001*** | 1.11 | 0.97; 1.28 | 0.130 |
| Low capacity (reference) |  |  |  |  |  |  |  |  |  |
| **Random effects** |  |  |  |  |  |  |  |  |  |
| Random intercept variance | 0.32 | 0.26; 0.38 | <0.001*** | 0.32 | 0.26; 0.40 | <0.001*** | 0.38 | 0.30; 0.47 | <0.001*** |

Abbreviation: aOR = adjusted odds ratio, CI = confidence interval

* *p*<0.05; ** *p*<0.01; *** *p*<0.001

a: 0 = poor or fair, 1 = excellent or good

b: 0 = definitely no, probably yes, probably no, 1 = definitely yes
